# Whole Exome Screening Identifies Novel and Recurrent *WISP3* Mutations Causing Progressive Pseudorheumatoid Dysplasia in Jammu and Kashmir-India

**DOI:** 10.1038/srep27684

**Published:** 2016-06-13

**Authors:** Ekta Rai, Ankit Mahajan, Parvinder Kumar, Arshia Angural, Manoj K Dhar, Sushil Razdan, Kumarasamy Thangaraj, Carol A. Wise, Shiro Ikegawa, Kamal Kishore Pandita, Swarkar Sharma

**Affiliations:** 1 Human Genetics Research Group, Department of Biotechnology, Shri Mata Vaishno Devi University, Katra, J&K, India; 2 School of Biotechnology, University of Jammu, Jammu, J&K, India; 3 Human Genetic Research and Counselling Centre, University of Jammu, Jammu, J&K, India; 4 7, Bhagwati Nagar, Jammu, J&K, India; 5 CSIR-Centre for Cellular and Molecular Biology, Hyderabad, Telangana, India; 6 Sarah M. and Charles E. Seay Center for Musculoskeletal Research, TSRHC; Departments of Orthopaedic Surgery and Pediatrics, and McDermott Center for Human Growth and Development, University of Texas Southwestern Medical Center, Dallas, Texas, USA; 7 Laboratory for Bone and Joint Diseases, RIKEN Center for Integrative Medical Sciences, RIKEN, Tokyo, Japan; 8 H.no:62, Lane 11, Swarn Vihar, Muthi, Jammu, J&K, India

## Abstract

We report identification and genetic characterization of a rare skeletal disorder that remained unidentified for decades in a village of Jammu and Kashmir, India. The population residing in this region is highly consanguineous and a lack of understanding of the disorder has hindered clinical management and genetic counseling for the many affected individuals in the region. We collected familial information and identified two large extended multiplex pedigrees displaying apparent autosomal recessive inheritance of an uncharacterized skeletal dysplasia. Whole exome sequencing (WES) in members of one pedigree revealed a rare mutation in *WISP3*:c.156C > A (NP_003871.1:p.Cys52Ter), that perfectly segregated with the disease in the family. To our surprise, Sanger sequencing the *WISP3* gene in the second family identified a distinct, novel splice site mutation c.643 + 1G > A, that perfectly segregated with the disease. Combining our next generation sequencing data with careful clinical documentation (familial histories, genetic data, clinical and radiological findings), we have diagnosed the families with Progressive Pseudorheumatoid Dysplasia (PPD). Our results underscore the utility of WES in arriving at definitive diagnoses for rare skeletal dysplasias. This genetic characterization will aid in genetic counseling and management, critically required to curb this rare disorder in the families.

Rare disorders are very often poorly understood in part due to their low prevalence and in the absence of specialized diagnostic tools and medical facilities. Such rare disorders may remain uncharacterized or misdiagnosed for years, even generations. This scenario is not uncommon in developing countries. We report identification and genetic characterization of a very rare skeletal disorder that remained undiagnosed for many generations, in a village of the Jammu and Kashmir region of India. In recent years the prevalence of the disorder has reportedly increased in the village, while the absence of any medical intervention and severity of the progressive skeletal disease has rendered affected individuals physically challenged and disabled for life. The village itself is located deep in the lower Himalayan mountains (approximate geographic coordinates: 33°47′17.9′N and 74°16′50.4′E) that until recently were inaccessible by modern transportation. The population residing in the region is highly endogamous and consanguineous with a size of approximately 4372 individuals (as per records from Ministry of Drinking Water and Sanitation, Government of India). Studies in the past have indicated that consanguineous marriages lead to increased chance of autosomal recessive disorders (reviewed in Hamamy 2012^1^) as also reported in the present study.

## Report

**Figure 1:**
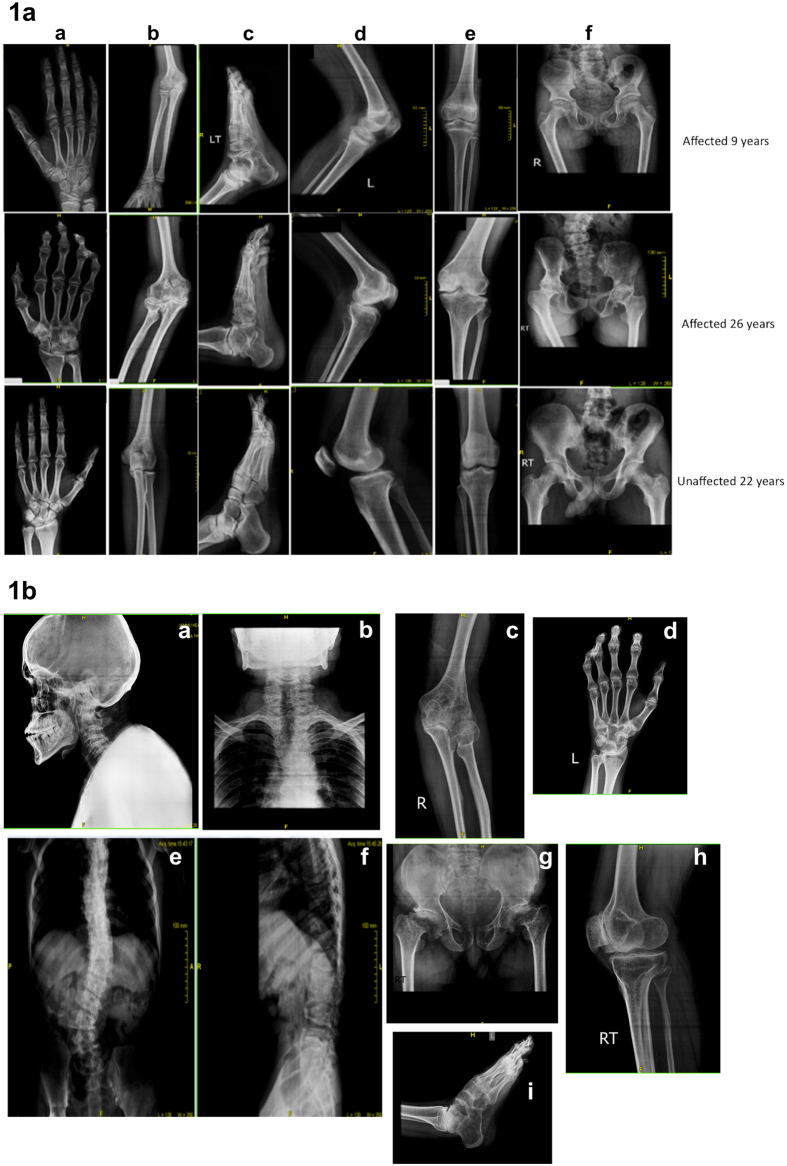
Representative Radiographs of Affected from both Families. (**1a)** Radiographs of Affected Sibs of Age 9 years and 26 years and Unaffected of Age 22 years. Panels show (a) Hand (b) Radial and Ulnar joints at Elbow (c) Feet (d) Knee Joint (lateral view) (e) Tibia and Fibula at knee joint (Anterior View) (f) Pelvis. (**1b)** Radiographs of affected individual of 32 years from 2nd family. Panels show (a) Head and Neck (lateral view) (b) Spine Thoracic region (posterior view) (c) Radial and Ulnar joints at Elbow (d) hand (posterior view) (e) Spine (posterior View) (f) Spine (lateral view) (g) Pelvis (h) Knee Joint (lateral view) (i) Foot (lateral view).

We collected familial information for the affected individuals and generated two highly extended multiplex pedigrees (supplementary Fig. 1a,b). The analyses of both pedigrees indicated very high consanguinity and an autosomal recessive mode of inheritance. We examined the phenotypic features of the disorder segregating within the two pedigrees. Affected individuals were reported to be normal at birth had normal intelligence, normal appearance and no symptoms of joint effusion or inflammation, with onset of disease symptoms between 4–6 years of age and intensifying with age. The disorder was reported to initiate with general physical symptoms including enlarged ankles, knees and elbow joints, knobby appearance of proximal interphalangeal joints of hand and mild involvement of hip joints. By ten years of age, in addition to the above symptoms, the affected appeared to develop a knobby appearance of metacarpophalangeal and distal interphalangeal joints of the hand and a slightly abnormal spine. In early adolescence, some affected individuals displayed gait disturbances due to knee varus, tilted pelvis and/or other spine deformities e.g. scoliosis. Beyond adolescence, affected individuals developed stiffness in the enlarged joints, comptodactyly in the fingers and toes, involvement of the shoulders, shortened stature by 10–18 cm due to dorsal kyphosis, a highly affected pelvis, and flexion contracture of the elbows and knees. Most individuals also presented with a chest deformity due to rib and spine involvement and they were unable to stand and extend their joints. The musculoskeletal features of the disorder warranted radiological screenings but facilities for this were lacking in the local village. We ultimately succeeded in transporting and an affected male (AR-11 of age 32 years) from the first family (Supplementary Fig. 1a) and two affected sisters (AR-17 of age 9 and AR-16 of age 26 years) and two unaffected members (IV-12, carrier father of age 60 and V-18, unaffected brother age 22 years) from second family (Supplementary Fig. 1b). In addition to a radiological examination the family members were evaluated by blood tests including: haemoglobin(Hb), erythrocyte sedimentation rate (ESR), blood sugar, calcium and alkaline phosphatase levels, presence of rheumatoid (RA) factor and anti nuclear antibodies (ANA). The results indicated values within normal ranges for all individuals. Radiological findings in the affected 9 year old sister showed enlarged epiphyses and metaphyses of the metacarpals and phalanges; enlarged femoral necks with reduced articular space of the hip joints; flexion of knees and reduced articular space in the knee joints; and reduced articular space in the feet (Fig. 1a). The older individuals (26 years, radiographs in Fig. 1a and 32 years, radiographs in Fig. 1b) exhibited additional radiological features including enlarged carpal bones with reduced interosseous space and comptodactyly; enlarged and flat femoral heads with complete loss of articular space in hip joints, significant osteopenia; and pelvic tilt due to abnormal curvature of the spine, complete loss of articular space in the knee joints and various spinal abnormalities (fused cervical vertebrae, narrowing of intervertebral disc spaces, kyphoscoliosis, platyspondyly, erosion of end plates and narrowing of intervertebral disc spaces) (Fig. 1a,b). Collectively these parameters indicate an inherited skeletal dysplasia with Mendelian recessive Inheritance. However, these clinical details were consistent with various diagnoses, making it difficult to precisely characterize the dysplasia.

**Figure 2:**
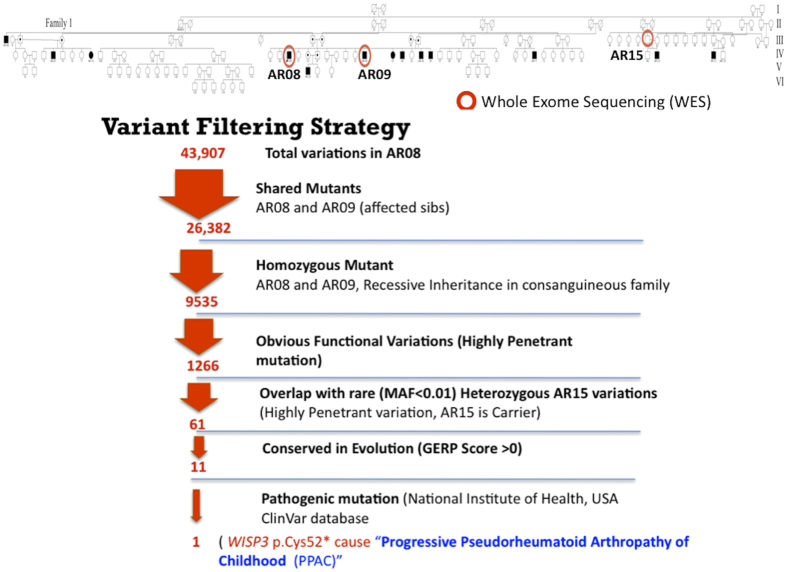
Whole Exome Sequencing and Variant Filtering Strategy adapted to narrow down to most promising causative mutation in Family 1.

Next generation sequencing (NGS), specifically whole exome sequencing (WES), has proven quite powerful for hastening the identification and characterization of rare Mendelian disorders^2^,^3^. Combining comprehensive WES, appropriate data filtering, and pedigree mapping can be sufficiently statistically informative to identify the single gene and causal mutation with as few as 2–4 family members members with very rare Mendelian disease^4^,^5^. We opted to carry out WES in the affected siblings from family 1 (Supplementary Fig. 1). We expected to observe a relatively high proportion of variants shared between these two individuals due to the consanguinity within the family rather than the disease, making it more difficult to distinguish true candidate variants. To partially overcome this issue and filter out false positives that would not segregate with the disease in the family we selected a distant cousin (AR-15 also a carrier) of the parents in family 1 (Fig. 2). We outsourced exome capture and sequencing to SciGenom Labs Private Limited. Blood samples were collected during the visit to the village and transported to lab in ice packs. DNA was isolated using Qiagen DNeasy Blood & Tissue Kit and approximately 2ug of genomic DNA and Agilent sureselect v5 kit for exome capture was used. Paired End (PE-100 × 2) Sequencing was done on Illumina HiSeq 2000 with a targeted mean coverage of 100X. A bioinformatics pipeline spanning from alignment variant calling to variant annotation was used (Details in SUPPLEMENTARY FILE Whole Exome Sequencing Methodology). The sequencing of the samples provided us over 90 million reads for each sample. More than 99% of the total reads aligned to the reference genome (GRCh37/HG19) for each sample. After filtering the alignment for mapping quality, 98% of the passed mapped reads have Q60 Phred score reflecting very good alignment quality. Variant calling was performed using BROAD Institute’s GATK-Toolkit^6^ and were called using the complete Human Reference Genome. Exome sequencing produced 26382 called variants that were shared in the affected individuals. After variant filtering for rare (<1%) conserved in evolution, functional homozygous recessive mutations, we identified the most promising causative variant in *WISP3*; NM_003880.3:c.156C > A NP_003871.1:p.Cys52*, which is listed in the USA National Institutes of Health ClinVar database^7^ (Fig. 2). This variation has been previously reported^8^,^9^ and is predicted to cause premature termination of the WISP3 protein. The mutation is diagnostic of Progressive Pseudorheumatoid Dysplasia (PPD), Progressive Pseudorheumatoid Arthropathy of Childhood (PPAC) or Spondyloepiphyseal Dysplasia Tarda with Progressive Arthropathy (SEDT-PA) (OMIM:208230). PPD is a very rare disorder with incidence estimated to be one case per million people in the UK^10^. The disorder has also been reported in India but primarily in Southern India^11^. We noticed, interestingly, the presence of another nonsynonymous variation *WISP3*; NM_003880.3:c.248G > A, NP_003871.1:p.Gly83Glu in the same exon. Both variations were checked for quality, and Sanger sequencing of the exon confirmed the finding (Supplementary Fig. 2a,b). Sanger sequencing was also performed on exon 2 of the *WISP3* gene in all the collected samples, with both variants showing perfect segregation with the disease in family 1 (Supplementary Fig. 3). However neither variant was detected by Sanger sequencing exon 2 in affected members of family 2. We further sequenced all the exons, exon-intron junctions and 5′-3′ UTRs of *WISP3* in family 2 and detected a novel splice site variant, NM_003880.3:c.643 + 1 G > A, at the junction of exon 4 and intron 4. This variant showed perfect segregation with the disease in family 2 (Supplementary Fig. 4). *In-silico* evaluation of the variant^12^ revealed that the site is highly conserved across the vertebrates (from humans to zebrafish). This change is predicted to alter the Exon 4 splice donor site (Supplementary Fig. 5), may be leading to either exon skipping/alternative splicing in the mRNA or premature termination of the protein, which needs to be experimentally validated. WISP3 is a member of the CCN gene family that encodes cysteine-rich secreted proteins with roles in cell growth and differentiation. It has been reported to modulate bone morphogenic protein (BMP) and the canonical WNT signaling pathway^13^. Earlier reports also note that homozygous or compound heterozygous mutations in the *WISP3* gene also cause PPD [reviewed in Garcia Segarra *et.al.*^14^].

In summary, the combined evidence of the segregation of *WISP3* functional variations with the disease, autosomal recessive transmission, and clinical and radiological features supported a diagnosis of PPD in both families. This identification and genetic characterization will aid in carrier screening and genetic counseling in both families. The ability to definitively identify PPD carriers and affected individuals may also help in managing the disorder, which ordinarily has a prevalence of one case per million people in the UK^10^ but thousands of fold higher prevalence in this isolated village. It was intriguing and highly unexpected to identify distinct causal mutations in the two families. Our results suggest that further screening of all affected individuals within the village and other members of these multiplex families is warranted to identify possible compound heterozygotes for these mutations. Further detailed medical evaluations are also needed to identify possible correlations between genotype and phenotype. It is critical for our future perspective to elucidate the functional consequence of the novel splice site variation we found (c.643 + 1 G > A) and its effect on the WISP3 protein to understand the mechanism of causation of the disease. This effort demonstrates the first genetic study exploiting WES to understand a disorder in the state of Jammu and Kashmir, India. WES may be an effective approach in understanding rare disorders in regions where such disorders exist in abundance but remain undiagnosed/misdiagnosed due to lack of specialized clinical resources.

### Ethics approval

 Institutional Ethical Review Board (IERB) SMVDU Katra, J&K, India approved the study and study was according to the approved guidelines. Informed consent for the study and genetic analyses was obtained from all individuals or their parents.

## Additional Information

**Accession Codes:** Next generation sequencing data (WES) will be available at SRA (NCBI) through Bioprojects; Project ids: PRJNA291671, PRJNA291678, PRJNA291679.

**How to cite this article**: Rai, E. *et al.* Whole Exome Screening Identifies Novel and Recurrent *WISP3* Mutations Causing Progressive Pseudorheumatoid Dysplasia in Jammu and Kashmir-India. *Sci. Rep.* **6**, 27684; doi: 10.1038/srep27684 (2016).

## Supplementary Material

Supplementary Information
