# Supplementary material for: Whole Exome Screening Identifies Novel and Recurrent WISP3 Mutations Causing Progressive Pseudorheumatoid Dysplasia in Jammu and Kashmir-India
Source: Sci Rep. 2016 Jun 13;6:27684. doi: 10.1038/srep27684 (PMC4904372; doi:10.1038/srep27684)
Supplement: Supplementary Information [file srep27684-s1.pdf]

Supplementary Figures and Data

Whole Exome Screening Identifies Novel and Recurrent *WISP3* Mutations Causing Progressive Pseudorheumatoid Dysplasia in Jammu and Kashmir India

Ekta Rai[1], Ankit Mahajan[2], Parvinder Kumar[3], Arshia Angural[1], Manoj K Dhar[2], Sushil Razdan[4], Kumarasamy Thangaraj[5], Carol Wise[6], Shiro Ikegawa[7], Kamal Kishore Pandita[8*], Swarkar Sharma[1*]

Authors' Affiliations:
1. Human Genetics Research Group, School of Biotechnology, Shri Mata Vaishno Devi University, Katra, J&K, India
2. School of Biotechnology, University of Jammu, J&K, India
3. Human Genetic Research cum Counselling Centre, University of Jammu, J&K, India
4. 7, Bhagwati Nagar, Jammu, J&K, India
5. Centre for Cellular and Molecular Biology, Hyderabad, A.P, India
6. Texas Scottish Rite Hospital for Children, Dallas, Texas, USA
7. Laboratory for Bone and Joint Diseases, SNP Research Center, RIKEN, Tokyo, Japan
8. Department of Internal Medicine, ASCOMS & Hospitals, Jammu, J&K, India

Corresponding Authors

Dr. Swarkar Sharma
Coordinator, Human Genetics Research Group
School of Biotechnology, Shri Mata Vaishno Devi University, Katra, J&K, India
Swarkar.sharma@smvdu.ac.in
Mobile: +91-9419955636 Ph: +91-1991-285535//285525 Ext. 2533, 2385

Dr. Kamal Kishore Pandita
Department of Internal Medicine, ASCOMS & Hospitals, Jammu, J&K, India
panditakk69@gmail.com

Running Title: Whole Exome Sequencing in Skeletal Dysplasia

Keywords: Whole Exome Sequencing, Skeletal Dysplasia, PPAC, SEDT-PA, PPD, India

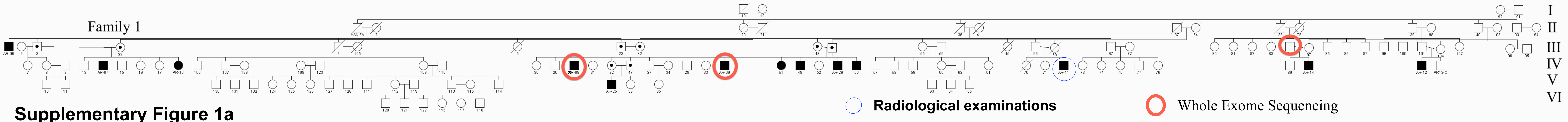

Supplementary Figure 1a

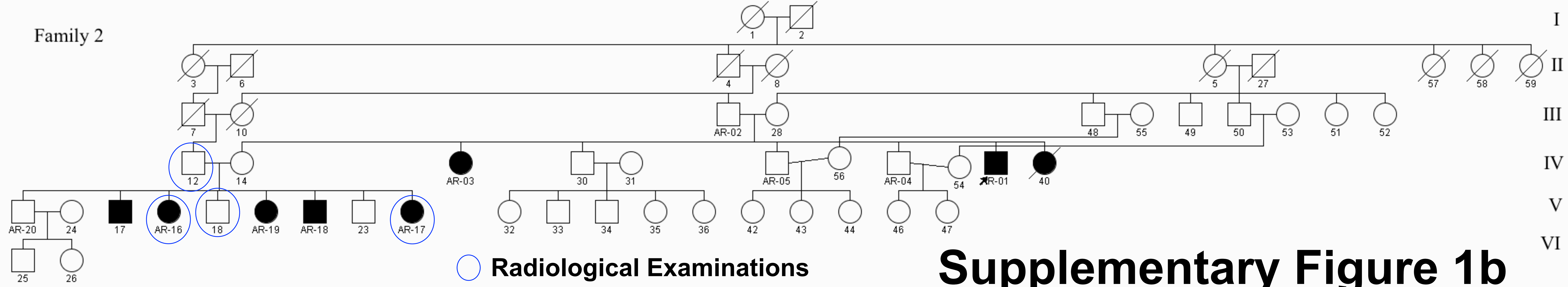

Supplementary Figure 1b

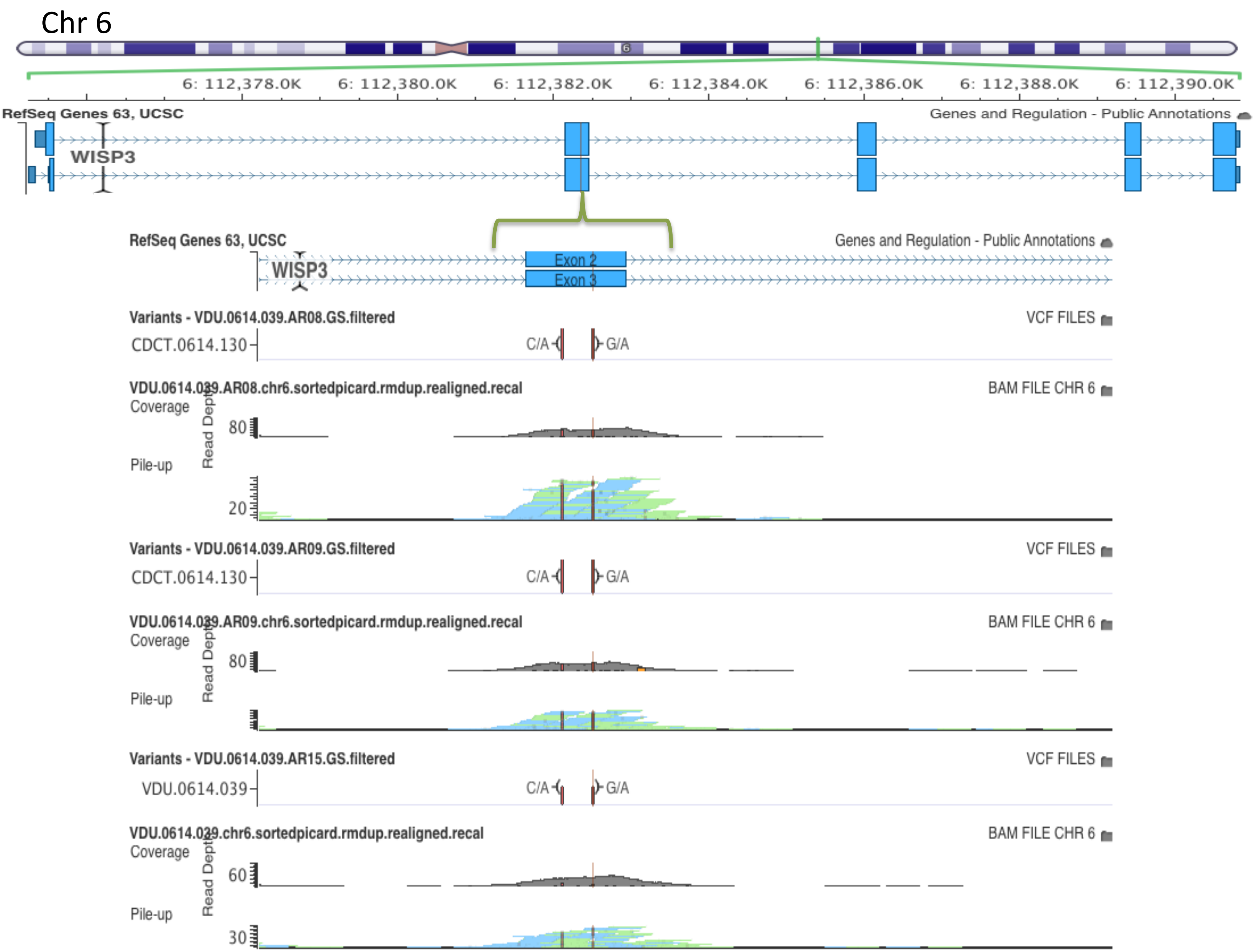

Supplementary Figure 2(a) Screenshot from Integrated Genome Browser (IGV, BROAD Institute) showing various quality parameters.

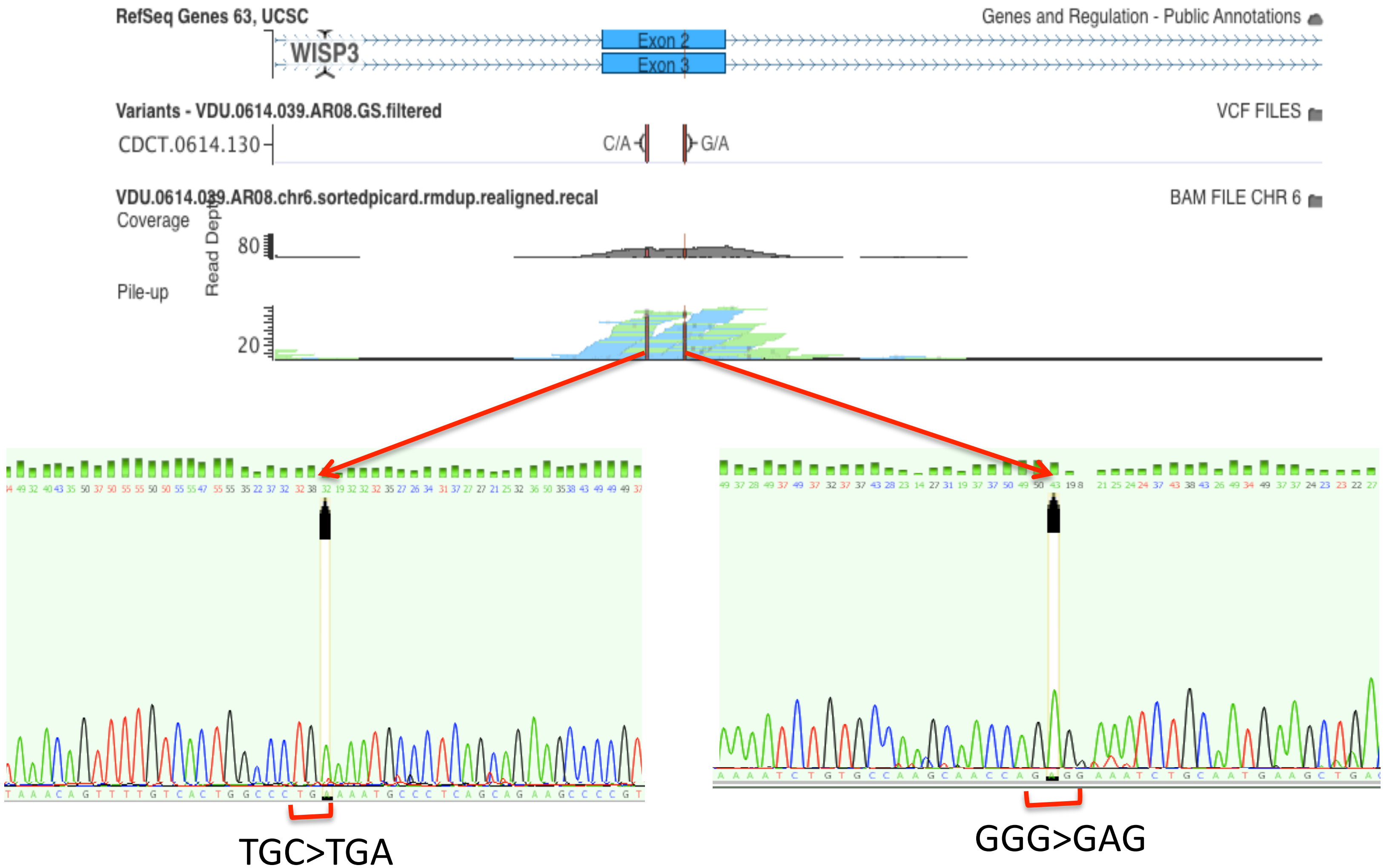

(RS121908901):
NM_003880.3:c.156C>A
NP_003871.1:p.Cys52*

(RS147337485)
NM_003880.3:c.248G>A,
NP_003871.1:p.Gly83Glu

Supplementary Figure 2(b): Sanger Sequencing Results of the region confirming both the variations.

VARIATIONS NP_003871.1:p.Cys52* (C>A) AND NP_003871.1:p.Gly83Glu (G>A) IN EXTENDED FAMILY 1

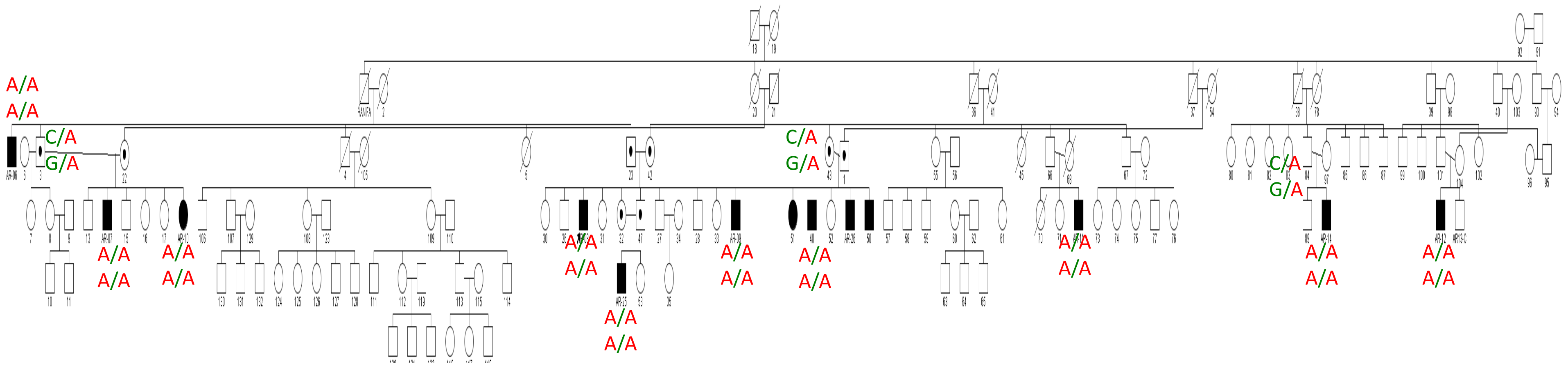

Perfect segregation with the disease in family

FAMILY 2

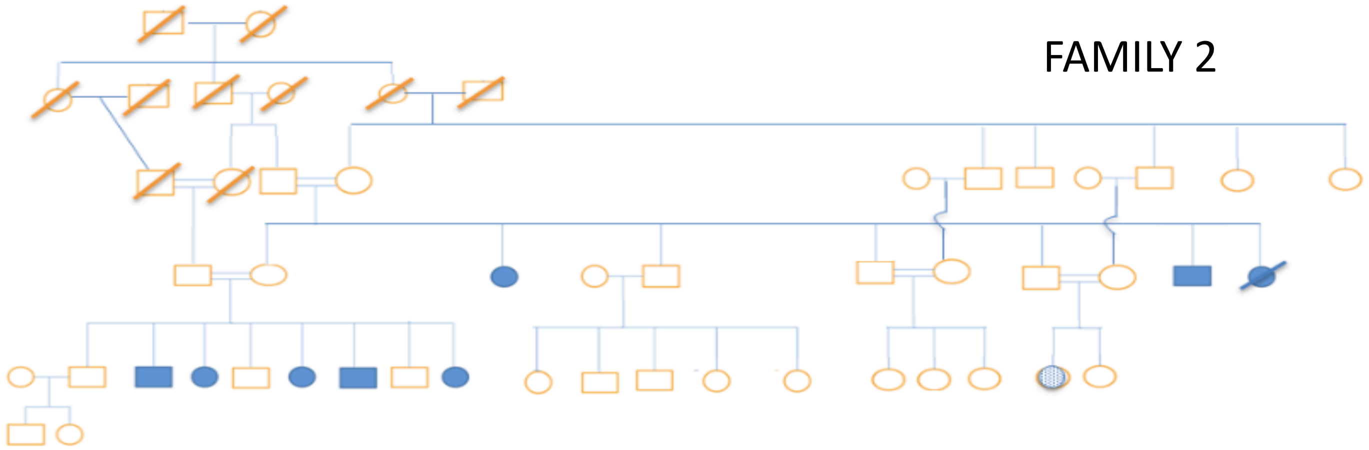

Both Variations were absent in 2nd Family.

Supplementary Figure 3: Variations WISP3 NM_003880.3:c.156C>A and NM_003880.3:c.248G>A distribution in Family 1 and 2

(Novel *WISP3* Splice site variation) NM_003880.3:c.643+1 G>A
IN EXTENDED FAMILY 2

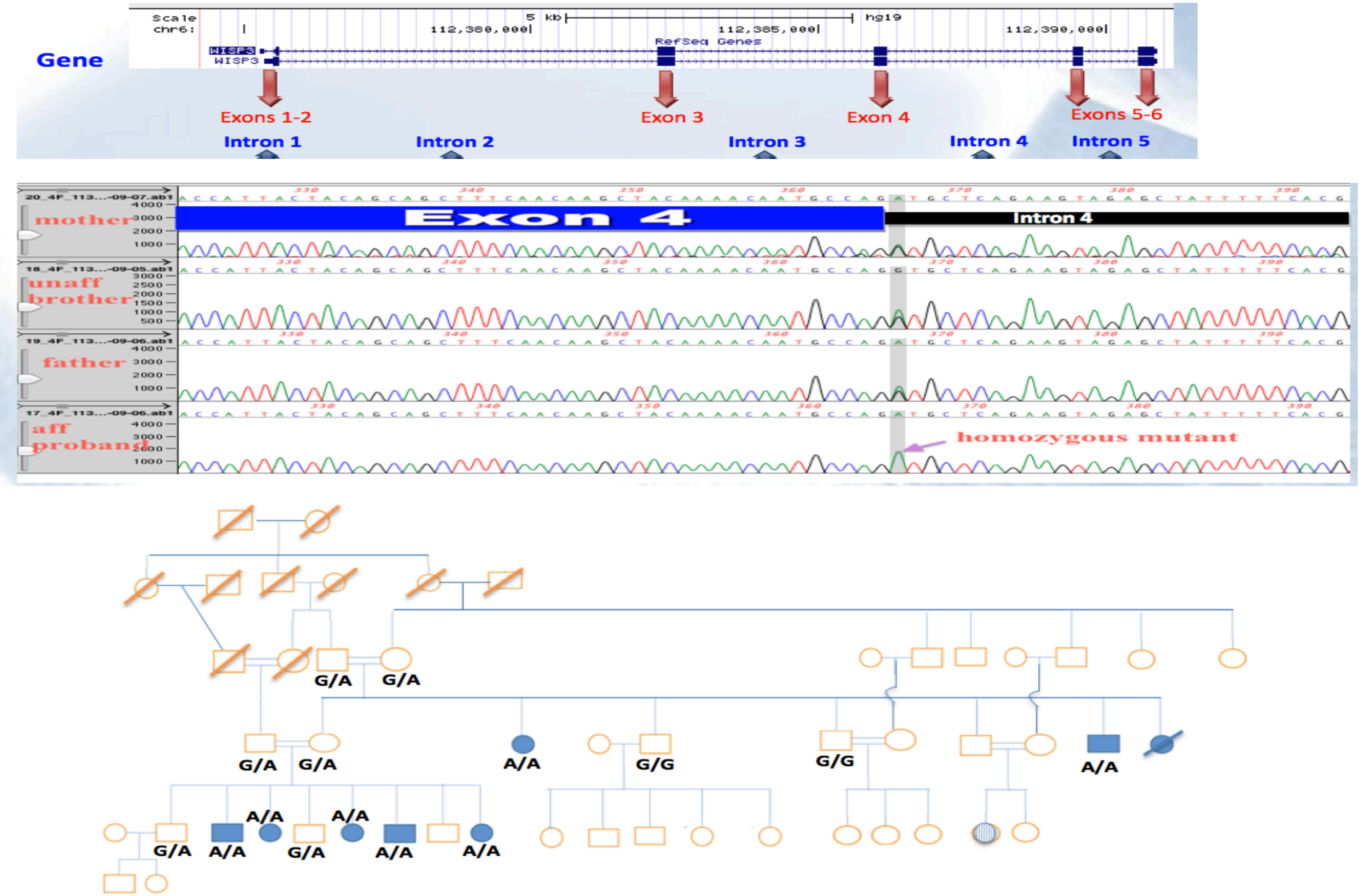

Perfect segregation with the disease

Supplementary Figure 4: Figure depicting Sequencing results at Exon 4 - Intron 4 Junction of WISP3 gene and distribution of the variation in Family 2.

(Novel *WISP3* Splice site variation) NM_003880.3:c.643+1 G>A

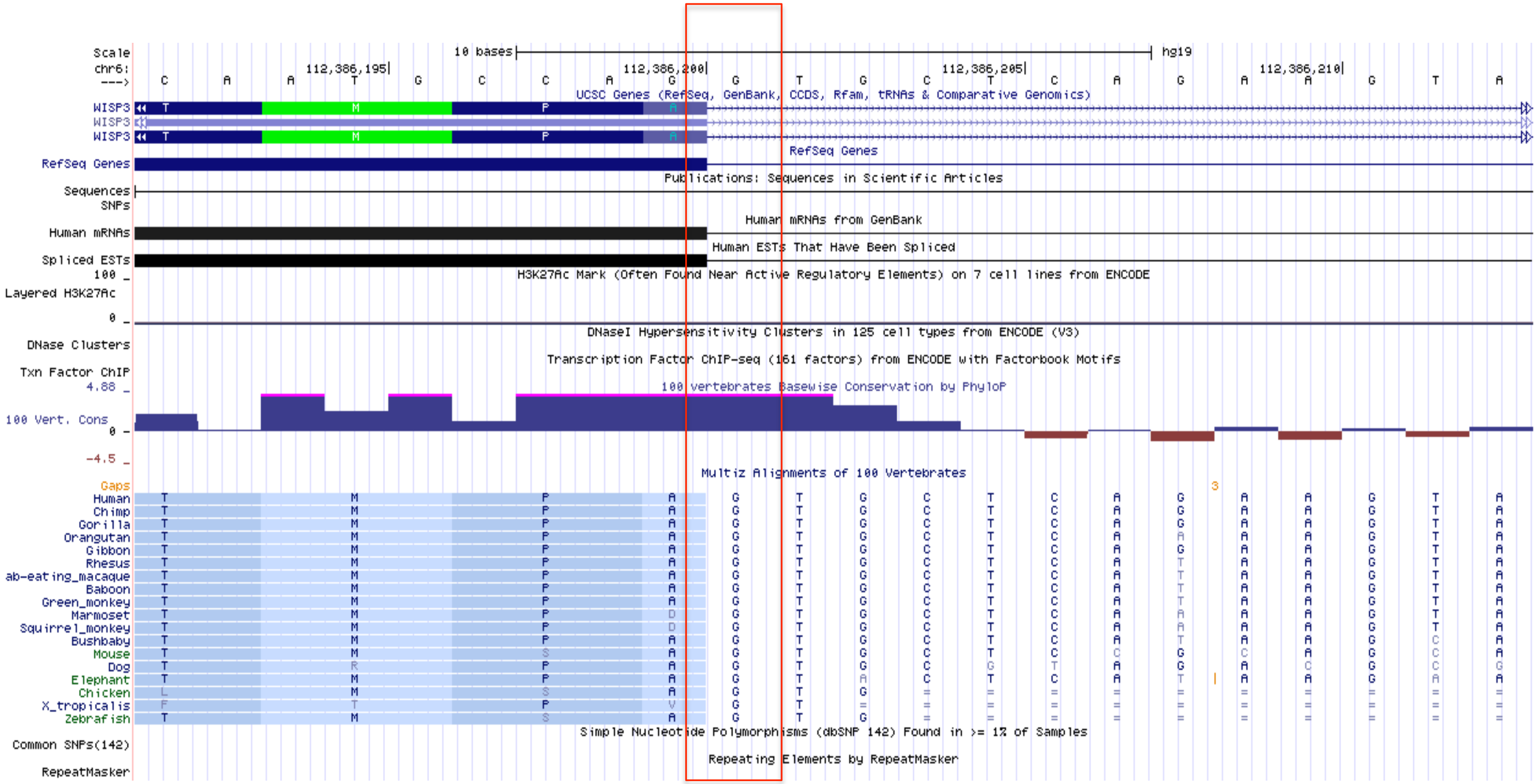

Predicted signal	Prediction algorithm	cDNA Position	Interpretation
Broken WT Donor Site	1 - HSF Matrices	T G C C A G g t g c t c a g a (1, 2; 238 240 242 244 246 248 250 252)	Alteration of the WT donor site, most probably affecting splicing.
	2 - MaxEnt		

FO Desmet, Hamroun D, Lalande M, Collod-Beroud G, Claustres M, Beroud C. Human Splicing Finder: an online bioinformatics tool to predict splicing signals. Nucleic Acid Research, 2009

Supplementary Figure 5: Screenshot from UCSC Genome Browser depicting conservation at the location across 100 Vertebrates and predicted effect of WISP3 Splice site variation NM_003880.3:c.643+1 G>A by Human Splicing Finder*

Supplementary Information Whole Exome Sequencing Methodology

Human Whole Exome Service was performed by SciGenom Labs Private Limited, Kakanad, Cochin, Kerala, India.

Exome sequencing

Agilent Sureselect v5 kit was used for the exon capture and sequencing was done on Illumina HiSeq 2000 with targeted mean coverage of 100X. A bioinformatics pipeline spanning from alignment, variant calling to variant annotation was used.

Bioinformatics analysis pipeline

The following bioinformatics steps were performed for preliminary analyses of the data.

Read quality check - We checked the following parameters from fastq file.

- Base quality score distribution
- Sequence quality score distribution
- Average base content per read
- GC distribution in the reads
- PCR amplification issue
- Check for over-represented sequences
- Adapter trimming

Based on quality report of fastq files, we trimmed sequence read where necessary to only retain high quality sequence for further analysis. In addition, the low-quality sequence reads were excluded from the analysis.

Read alignment – The paired-end reads were aligned to the reference human genome Feb. 2009 release downloaded from UCSC database (GRCh37/hg19). The chromosome fasta file was downloaded from the following website (http://hgdownload.soe.ucsc.edu/goldenPath/hg19/bigZips/chromFa.tar.gz). The alignment was performed using BWA program (version= 0.7.0-r313). Only properly paired aligned reads (based on flagstat summary of samtools) and reads with mapping quality >= 30 were taken further for downstream analysis.

Duplicate read removal – The aligned reads were first sorted using Picard tool (version 1.85) Sort Sam command and then the read duplicates were removed using Picard MarkDuplicates command.

Read realignment– After removing duplicate reads the reads were realigned around the known indels from 1000-genome study using GenomeAnalysisTKLite-2.3-9 toolkit.

The known indels set (Mills_and_1000G_gold_standard.indels.hg19.vcf) was downloaded from GATK resource page.

Base recalibration – After performing realignment the base re-calibration step was performed. After recalibration, the quality score of each base was more accurate. For details please look into the following website http://gatkforums.broadinstitute.org/discussion/44/base-quality-score-recalibration-bqsr. Known variant position (dbsnp_138, Mills_and_1000G_gold_standard) was taken into account to recalibrate the quality score.

Variant calling – After performing realignment, we used GenomeAnalysisTKLite-2.3-9 toolkit UnifiedGenotyper to identify single nucleotide variants (SNPs) and short Indels. After calling the variants, we further filtered variants in order to retain good quality (depth, variant score and others) variants.

Variant annotation and comparison – The identified variants were annotated using in-house program (VariMAT) at SciGenom Pvt. Ltd. The gene model used for annotation was downloaded from Ensembl database Release 75 (ftp://ftp.ensembl.org/pub/release-75/gtf/homo_sapiens).

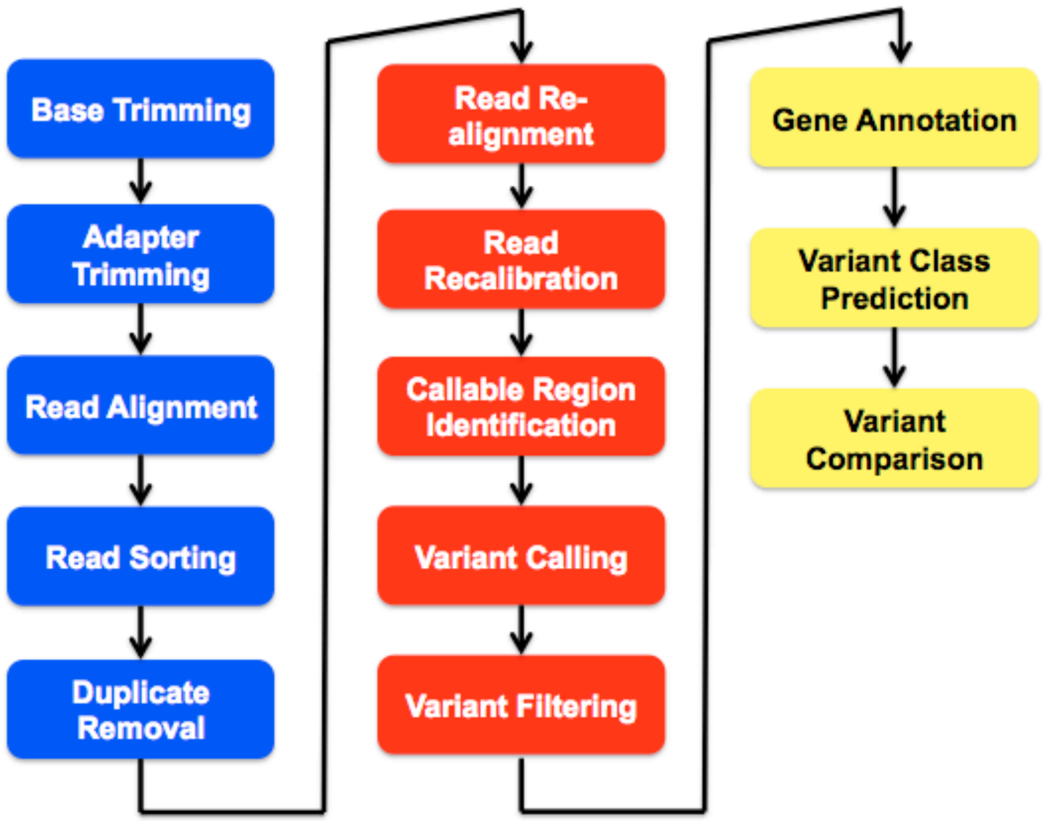

Supplementary Figure 6: Bioinformatics analysis pipeline

Analysis results.

Data summary

The sequencing of the sample on Illumina HiSeq 2000 provided us more than 90 million reads for each sample. Total data generated for the each sample was more than 8 Gb. Approximately around 90% of data was greater than or equal to Q30 Phred score.

Alignment & Filter

The overall alignment summary is shown in Table 1. Overall, more than 99% of the total reads aligned to the reference genome for each sample. After filtering the alignment for mapping quality, insert size estimates and cross mapping read more than 96% passes the alignment filer. More than 98% of the passed mapped reads in all samples have Q60 score, which reflects very good alignment quality (Supplementary Figure 7). Insert size distribution from filtered aligned reads, is shown in

Supplementary Figure 8. The percentage duplicate reads per chromosome is shown in Supplementary Figure 9. Less than 1% of reads out of aligned reads are duplicated in the chromosomes for all three samples.

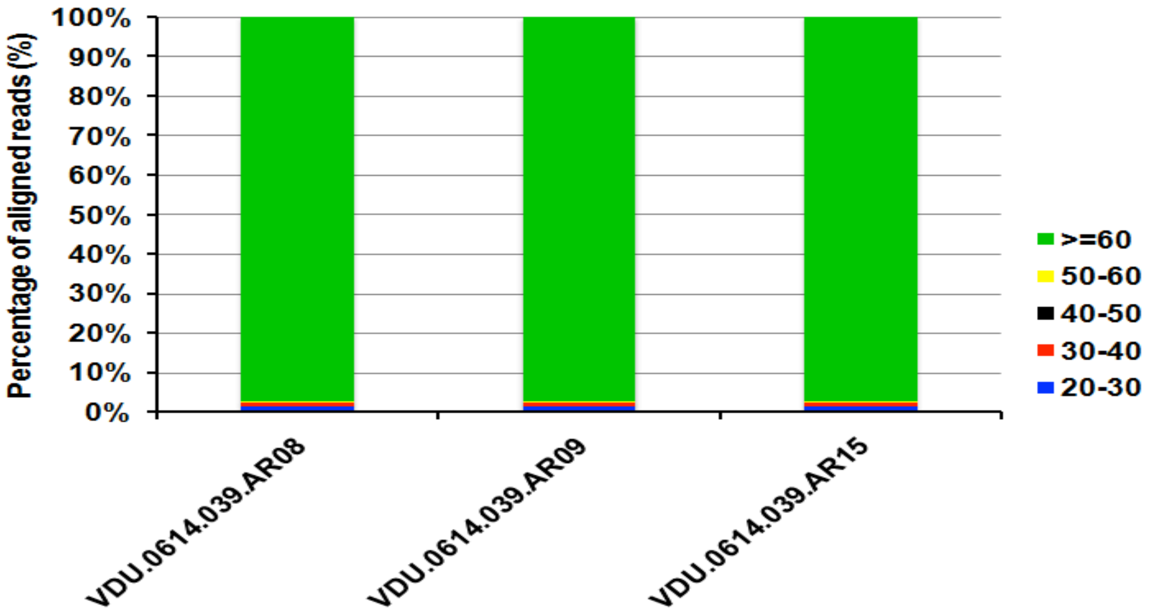

Supplementary Figure 7: Mapping quality distribution for aligned reads

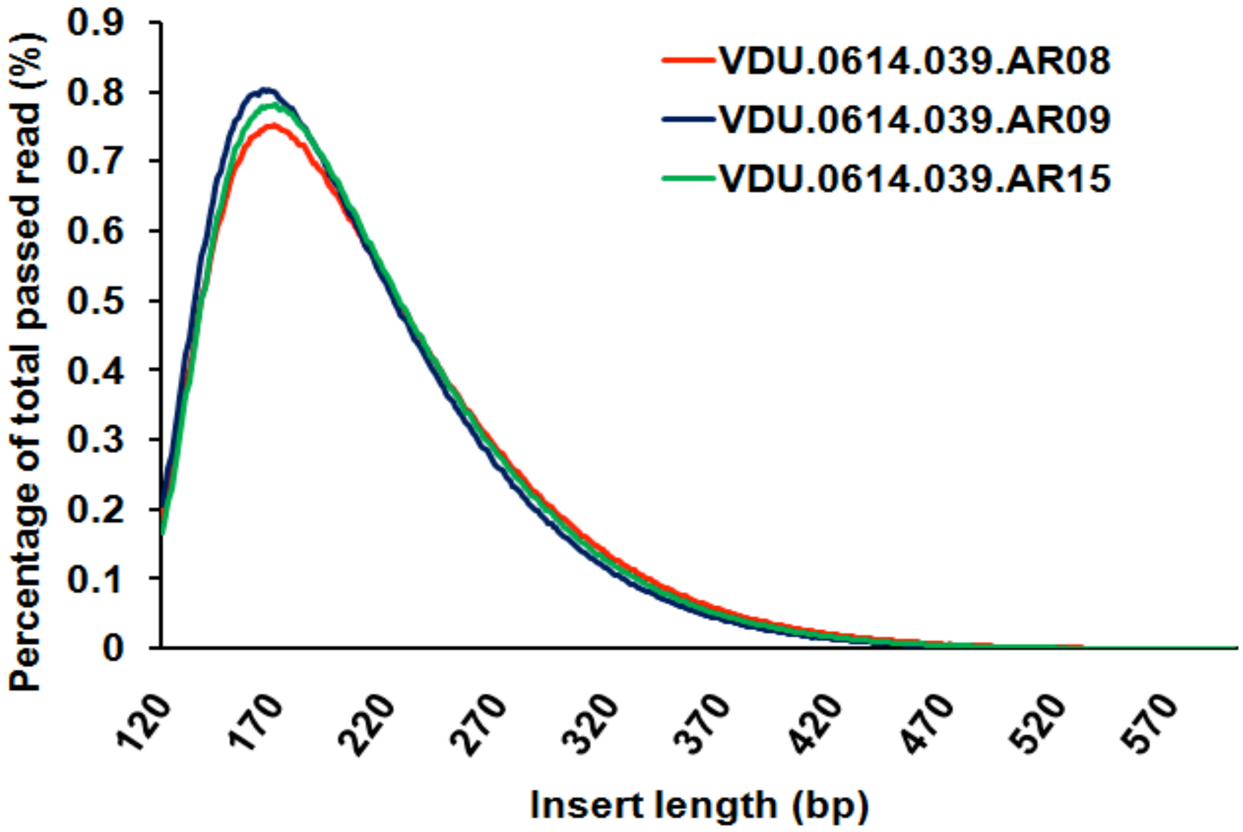

Supplementary Figure 8: Insert-size distribution of the aligned reads

Table 1: Read alignment summary

	Total Reads	Total aligned (%)	Total passed alignment (%)	Total Failed alignment (%)
**VDU.0614.039.AR08**	90,333,846	99.54978669	96.90474709	2.493195076
**VDU.0614.039.AR09**	93,840,398	99.56829893	96.58313257	2.744785886
**VDU.0614.039.AR15**	87,287,262	99.71901055	96.85005929	2.619693811

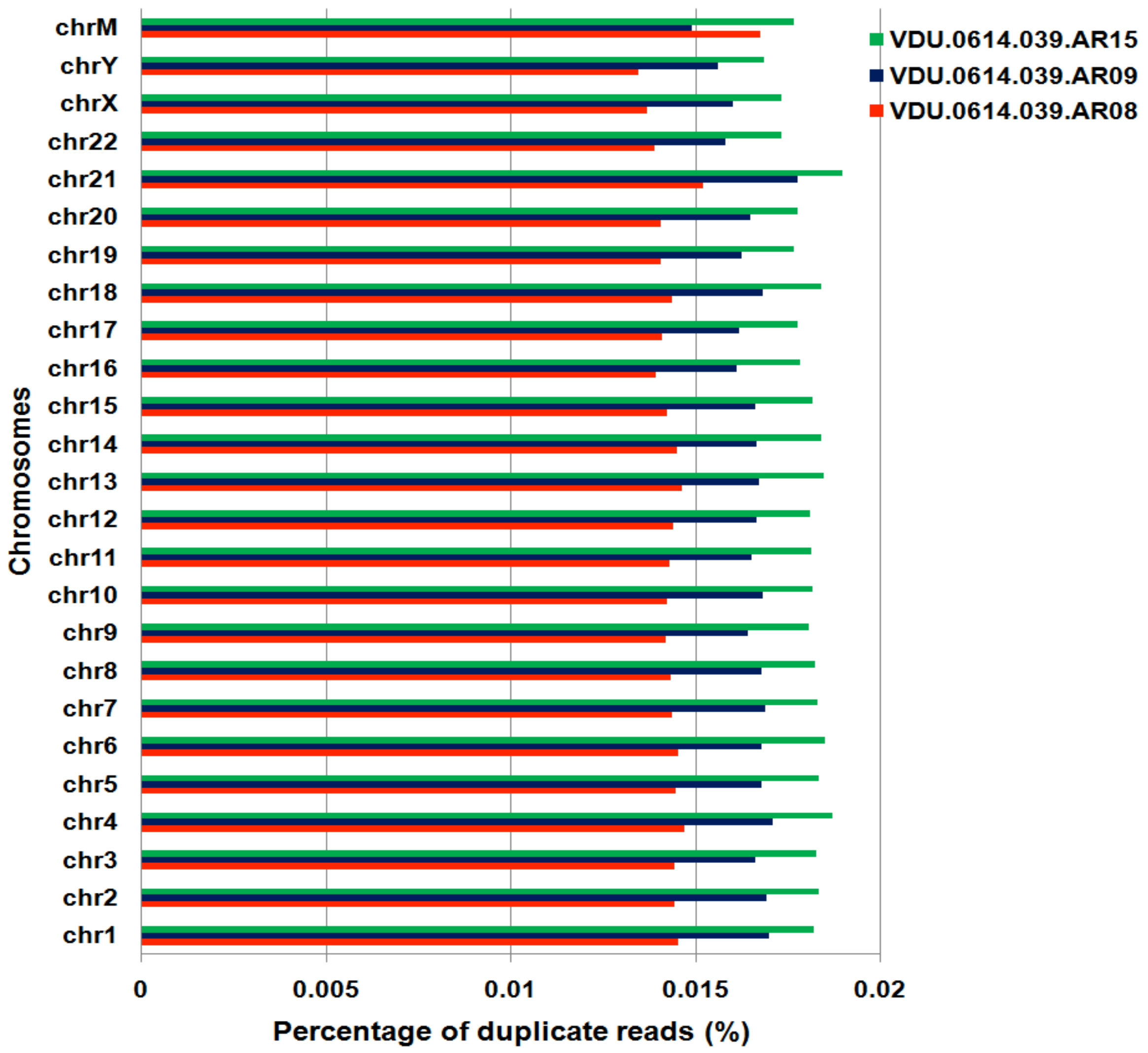

Supplementary Figure 9: Percentage duplicate reads per chromosome

Variant calling

The variant calling was performed using GATK-Lite program. After identification the variants were filtered out. The main criteria for accepting the variants as good quality were: Min. Read Depth >= 10; Variant Quality >= 50; Variant not in SNP cluster; Multi-mapped read< 5. The variants were called using the complete reference genome, later we filtered the variants only for the targeted genomic regions – based on the kit used (Agilent Sure Select V5) library preparation. The filter passed variants are provided in Table 2. The variant depth, quality and variant minor allele frequency distribution is provided in Supplementary Figure 10-12 respectively. The indel base distribution is provided in Supplementary Figure 13. For majority of the indels 1-bp was either deleted or inserted with respect to the reference genome.

Table 2: Summary of variant identified

	VDU.0614.039.AR08	VDU.0614.039.AR09	VDU.0614.039.AR15
**TOTAL VARIANTS**	43,907	44,350	42,937
**TOTAL SNPs**	40,795	41,174	39,896
**TOTAL INDELs**	3,112	3,176	3,041
**TOTAL HET**	26,300	26,739	24,979
**TOTAL HOM**	17,607	17,611	17,958
**Ts/Tv**	2.593127753	2.583543753	2.571594878

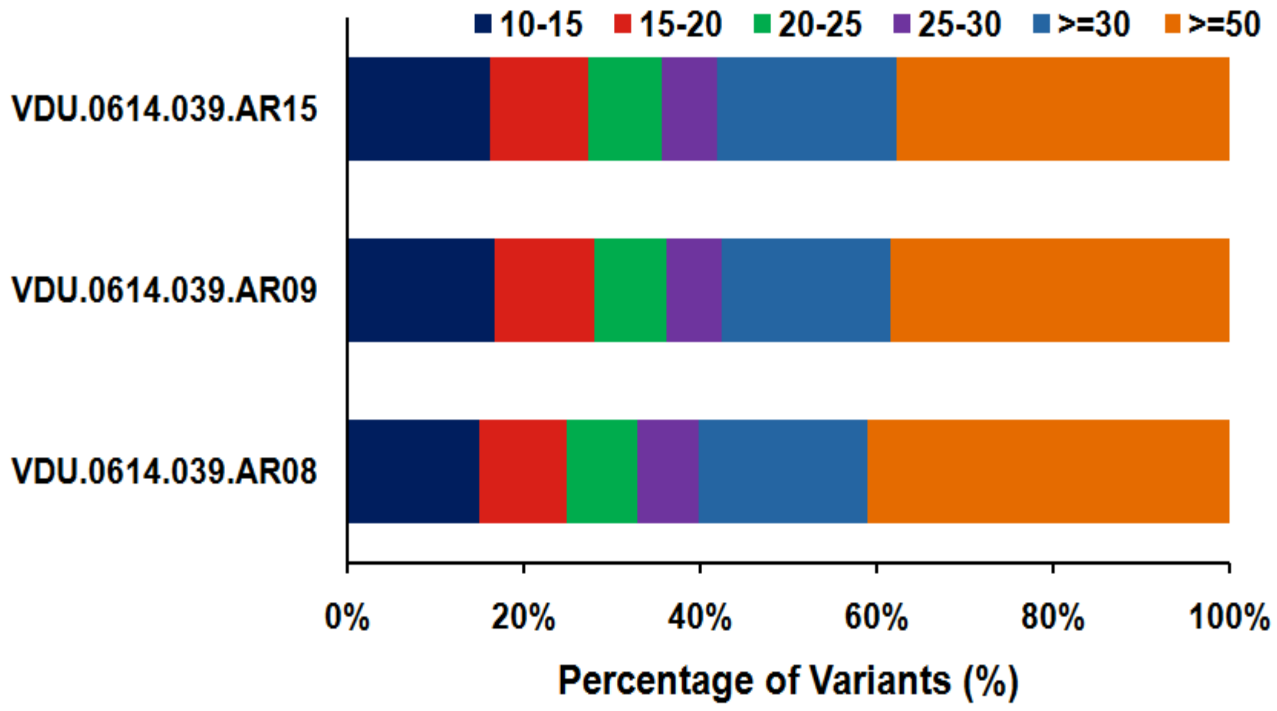

Supplementary Figure 10: Read depth distribution of the identified variants in the sample. The legend represent variant read depth

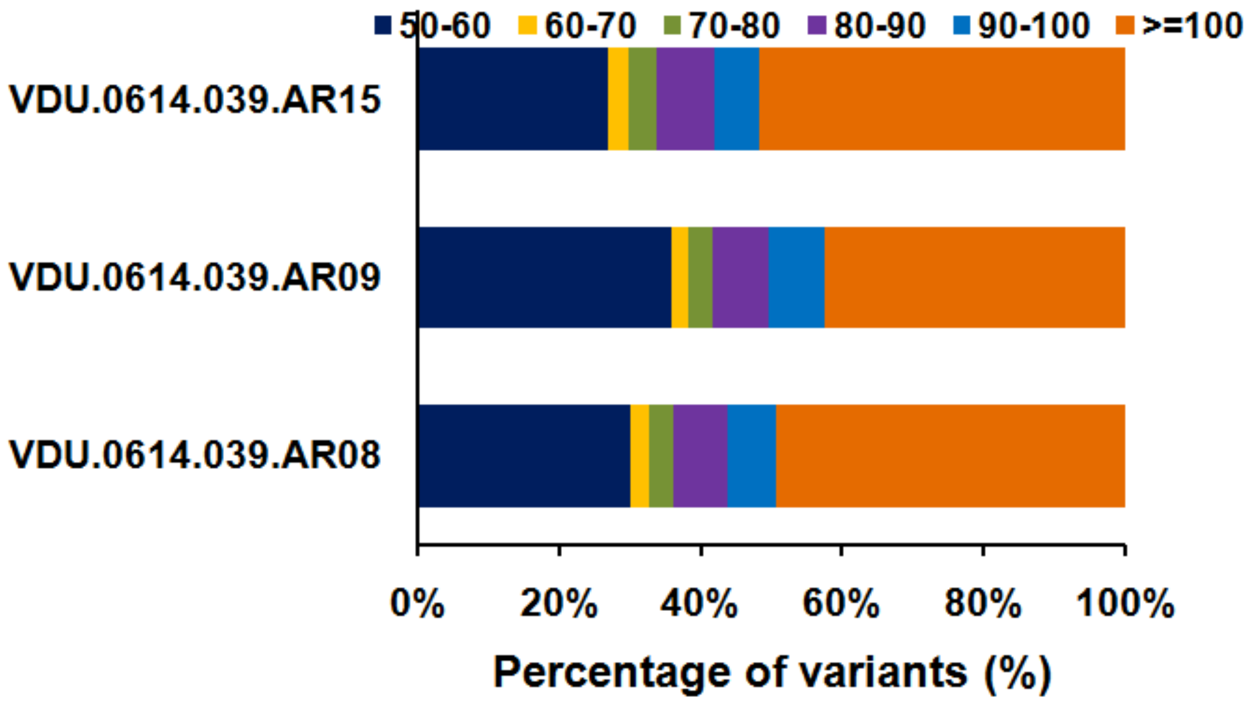

Supplementary Figure 11: Quality distribution of the identified variants in the sample. The legend represents variant quality score

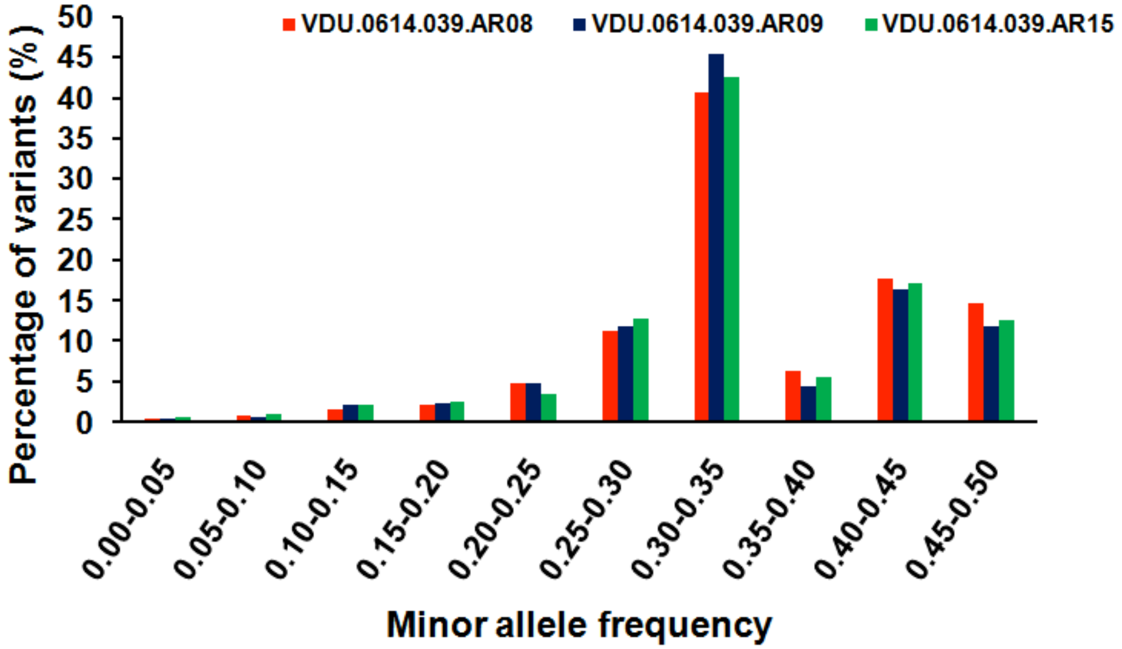

Supplementary Figure 12: Variant minor allele frequency distribution for all samples

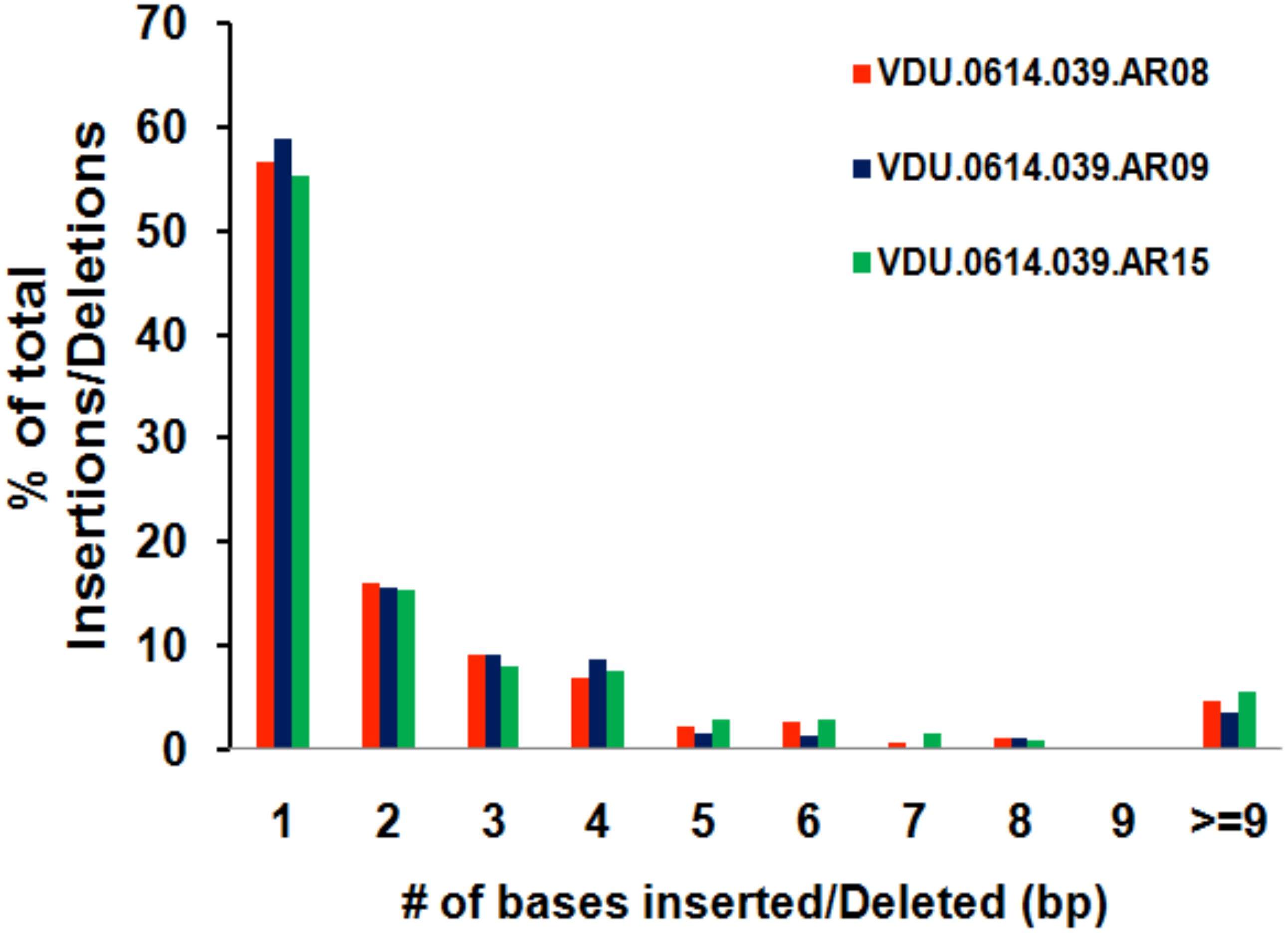

Supplementary Figure 13: Distribution of insertion and deletion base length

Variant annotation

The identified variants were annotated and annotation summary of filtered SNPs and Indels based on the targeted genomic regions (based on the kit used for the library preparation) is provided in Tables 3 and shown in Supplementary Figure 14. Out of total variants identified, ~98% were present inside gene region. Approx. 63% of the SNPs and Indels fall in the exonic region. The total variants identified in different variant classes are provided in Table 4, also shown in Supplementary Figure 15.

Table 3: Annotation summary of variants

	VDU.0614.039.AR08	VDU.0614.039.AR09	VDU.0614.039.AR15
**Intergenic**	547	497	560
**InsideGene**	43,360	43,853	42,377
**Exonic**	27,632	27,833	26,976
**Intronic**	15,728	16,020	15,401
**Exonic-CDS**	20,797	21,057	20,328
**Exonic-5UTR**	1,194	1,262	1,151
**Exonic-3UTR**	1,582	1,567	1,551
**Exonic-PC_ncRNA**	2,573	2,501	2,510
**Exonic-ncGene**	1,486	1,446	1,436
**Intronic-3SPLICE_SITE**	1,076	1,093	1,036
**Intronic-5SPLICE_SITE**	817	813	804
**Intronic-Others**	13,835	14,114	13,561

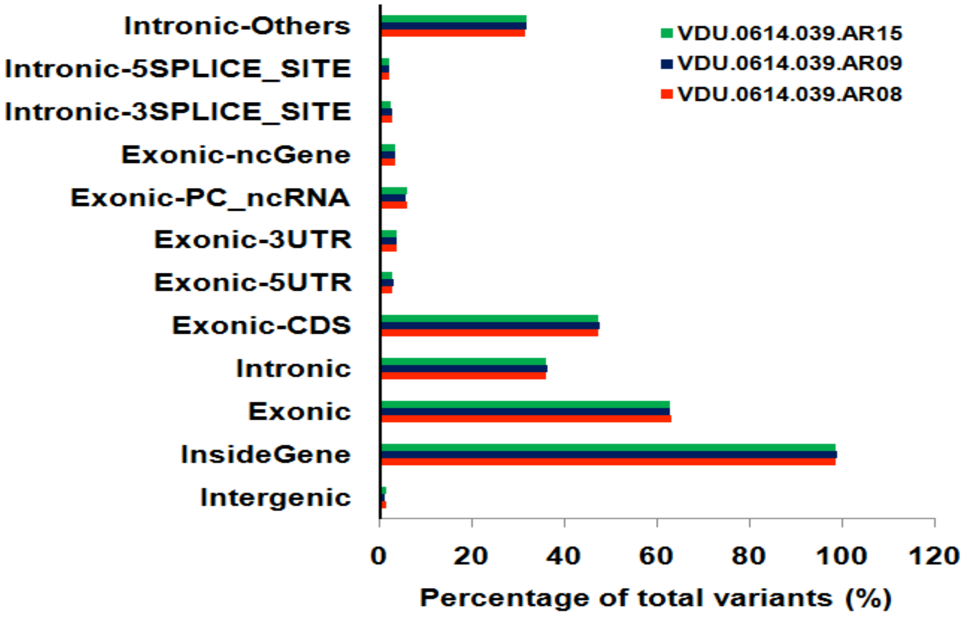

Supplementary Figure 14: Distribution of all identified SNPs and Indels in the samples

Table 4: Functional prediction of coding variants

VariantClass	VDU.0614.039.AR08	VDU.0614.039.AR09	VDU.0614.039.AR15
**Silent**	9,318	9,446	9,066
**Missense**	10,776	10,904	10,578
**Nonsense**	182	182	169
**Startloss**	30	27	34
**Stoploss**	99	105	96
**Frameshift-indel**	204	219	204
**Inframe-indel**	182	171	178

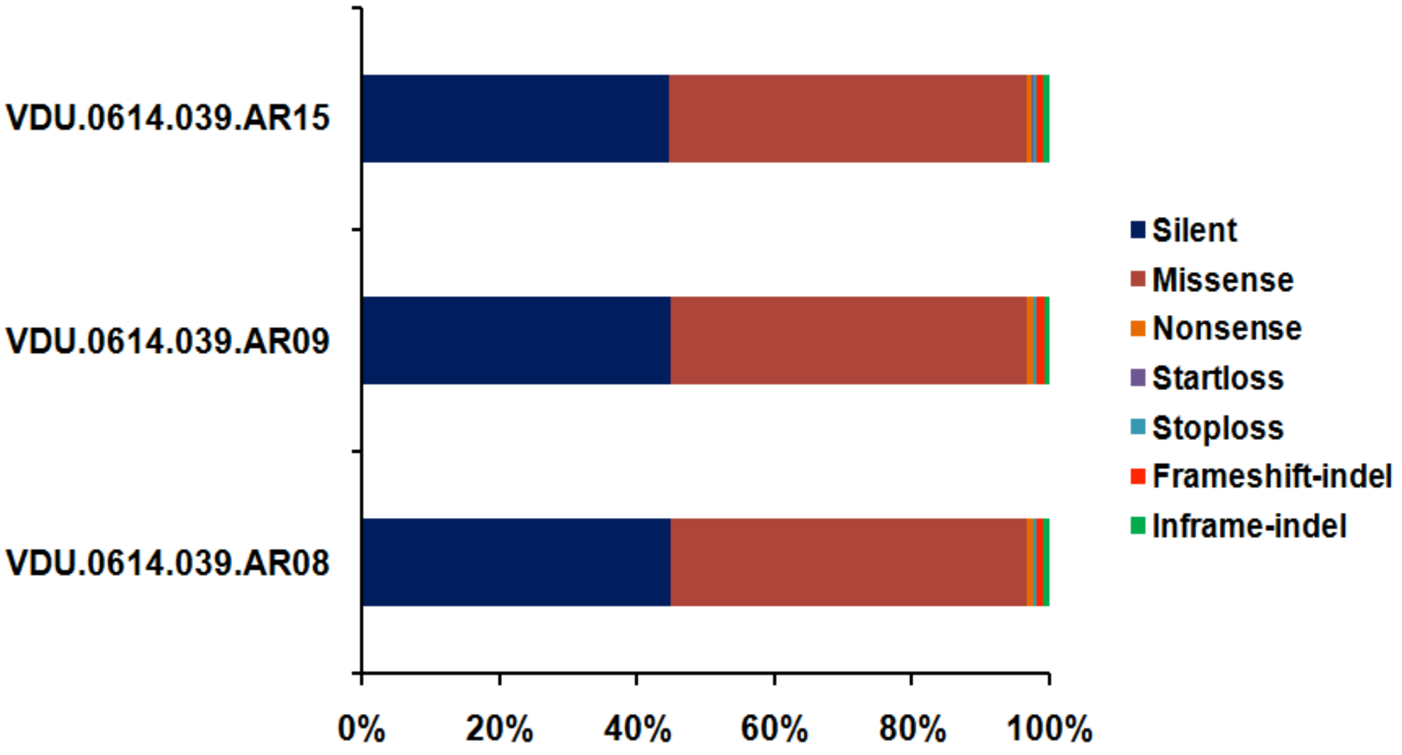

Supplementary Figure 15: Distribution of coding variants
